# An unusual cause of haemoptysis in childhood: A case report and literature review

**DOI:** 10.7196/SARJ.2018.v24i3.191

**Published:** 2018-09-07

**Authors:** S Chaya, R de Decker, M Zampoli, A Vanker

**Affiliations:** 1 Department of Paediatrics and Child Health, Division of Paediatric Pulmonology, Red Cross War Memorial Children’s Hospital and University of Cape Town, Cape Town, South Africa; 2 Department of Paediatrics and Child Health, Division of Paediatric Cardiology, Red Cross War Memorial Children’s Hospital and University of Cape Town, Cape Town, South Africa

**Keywords:** broncho-oesophageal fistula, haemoptysis

## Abstract

Haemoptysis is uncommon in children and the diagnosis is challenging. We describe a 14-year-old child who presented with haemoptysis secondary to a suspected congenital broncho-oesophageal fistula. This is a rare condition and the symptoms are insidious, occasionally beginning in childhood but may present only in adulthood. The case report describes the presentation, diagnosis and management of broncho-oesophageal fistulas, with a review of the current literature.

## Background

Haemoptysis is uncommon in children, although there are numerous causes. These include bronchiectasis, congenital heart disease, infections, alveolar haemorrhage and neoplasms.^[1]^ Symptoms range from mild to severe. The diagnosis is often challenging, as children swallow their sputum and the haemoptysis often goes unnoticed. Haemoptysis is classified as massive or non-massive. Massive haemoptysis is considered if blood loss is estimated at more than 200 mL per day, which should be elicited from a thorough history.^[1]^ We present an unusual case of haemoptysis due to a suspected congenital broncho-oesophageal fistula.

## Case

AL is a 14-year-old male patient with cerebral palsy. He presented with a 3-day history of cough and haematemesis; the volume quantified was ~125 mL. He was previously treated at a primary healthcare centre for pulmonary tuberculosis (TB). The TB diagnosis was based on prolonged coughing and suggestive radiological changes, but it was not confirmed microbiologically (Gene Xpert or culture confirmation). The chest radiograph from the peripheral hospital was unavailable.

Clinically he was apyrexial and haemodynamically stable. He was not pale, had no clubbing and his chest examination was normal, with only epigastric tenderness noted. He was treated with omeprazole for acute gastritis, with a non-urgent gastroscopy planned for the future. The chest radiograph demonstrated an irregular right-sided hilar opacity.

At the time of discharge, he had a further significant episode of haemoptysis with vomiting. Approximately 500 mL of blood was noted. He remained haemodynamically stable, but his haemoglobin dropped from 9.1 g/dL to 8 g/dL. Coagulation parameters, including platelets, were normal. A repeat chest radiograph was unchanged from the initial one. Repeated sputum analyses for Gene Xpert and TB culture were negative.

A gastroscopy demonstrated a dilated and abnormal oesophagus with an ulcer and an oesophageal biopsy was in keeping with mild oesophagitis. On bronchoscopy, the airway anatomy was normal; a fistula opening was not seen. There was also no evidence of pulmonary haemorrhage or any endobronchial lesions, but purulent secretions were noted to arise from the right lower lobe segments.

**Fig. 1:**
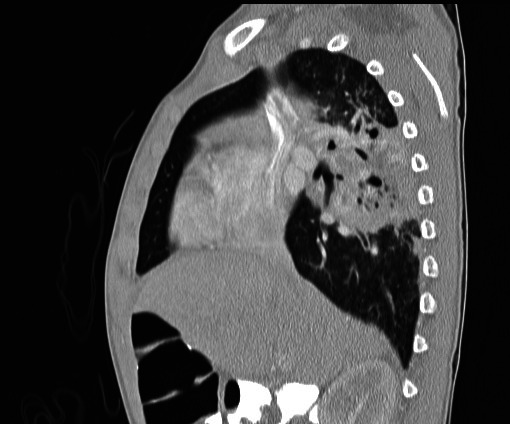
Computed tomography image with the mass-like opacification in the superior segment of the right lower lobe, and in close proximity to the oesophagus.

A computed tomography (CT) scan of the chest showed an irregular contrast-enhancing inflammatory mass, with cavities located in the superior segment of the right lower lobe (Fig. 1).

**Fig. 2:**
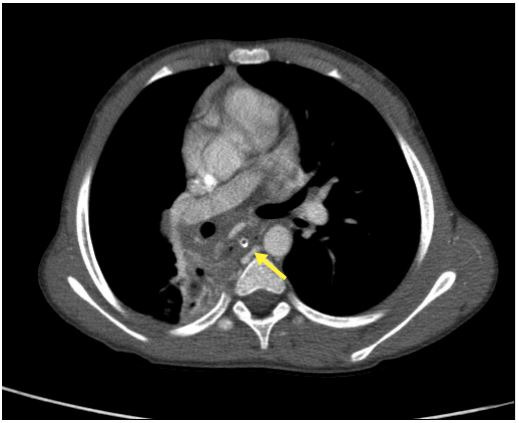
Computed tomography image demonstrating the complex vascular supply to the mass. Arrow indicates the branch of the aorta supplying the mass.

Two large arteries originating from the right lateral aspect of the descending aorta were noted to supply this mass, with another arterial supply by the bronchial artery (Fig. 2).

 The upper (more cranial) artery divided: one branch supplied the mass and the second ran superior and posterior and was thought to represent the artery of Adamkiewicz (also known as the great anterior segmental medullary artery).

An upper gastrointestinal contrast study confirmed the presence of a fistulous tract between the oesophagus and the cavities within the mass and the right lower main bronchial segments. There was also severe gastro-oesophageal reflux. Given this configuration, the diagnosis of a complex hybrid congenital pulmonary airway malformation with a broncho-oesophageal fistula (BOF) was considered. In view of the complexity of the vascular supply, surgery was deemed unsafe. As the child had ongoing bleeding, he was referred to the Cardiology department for embolisation of the systemic arteries supplying the lesion. One Amplatzer vascular plug (type II) was deployed into each artery, with obliteration of the blood supply to the lesion. Care was taken to not occlude flow to the (possible) artery of Adamkiewicz, as that could result in an anterior spinal artery syndrome. Oral feeds were suspended and a Nissen fundoplication and percutaneous endoscopic gastrostomy was performed to reduce reflux and gastropulmonary aspiration and allow inflammation to subside.

At follow-up 5 months after discharge, AL was doing well. He had no further episodes of haemoptysis or vomiting. A chest radiograph showed a considerable reduction in mass size.

His barium swallow showed a marked reduction in the fistula size and volume of contrast entering the lesion. There was no evidence suggesting any spinal vascular compromise after occlusion of the arteries feeding the mass. The long-term plan is to continue conservative management and allow the fistula to heal spontaneously. Failure of spontaneous closure will require surgery to excise the mass and remove the fistula.

## Broncho-oesophageal fistulas

Congenital BOFs were first reported by Gibson in 1696, and again by Negus in 1929.^[2]^ This is a rare condition and most documented cases have been isolated case reports. The largest published series included 100 patients, 24 of whom were children.^[3]^ BOFs occur equally in male and female patients. Age of presentation is variable: the youngest patient was 9 days old while the oldest was an 83-year-old man.^[3]^

Four types of congenital BOF are described.^[3]^ Type 1 has a wide-necked congenital diverticulum of the oesophagus, with an inflammatory fistula at the tip. Type 2 is the simplest, with a short track that runs from the oesophagus to the lobar or segmental bronchus. In type 3, a fistulous track connects the oesophagus to a cyst in the lobe that communicates with the bronchus. In type 4, the fistula communicates with a sequestered segment, which is identified by the presence of a branch of a systemic artery from the aorta. The latter, type 4, describes the fistula of our patient.^[3]^

BOFs have either a congenital or an acquired cause. Congenital causes include isolated malformations or are in association with other anomalies such as bronchopulmonary sequestration, which is the most common.^[4]^ Acquired causes include infections (*Mycobacterium tuberculosis*, histoplasmosis, actinomycosis), trauma, ingestion of caustic materials or inhalation of a foreign body.^[4]^ Unlike in adults, most acquired causes are non-malignant in children. Acquired BOFs secondary to tuberculosis should always be considered, especially when symptoms occur in older children and the child resides in an area where TB is highly endemic, as was the case with our patient.^[4]^ Differentiating a congenital BOF from an acquired one is difficult, especially in the presence of advanced pulmonary disease. This is definitively diagnosed only on histological examination by the absence of surrounding inflammation, the absence of adherent lymph nodes and the presence of a mucosa and muscularis mucosa.^[5,6]^

The most common location of BOFs is between the middle third of the oesophagus and the right lower lobe (41%), followed by the left lower lobe (21%), right main bronchus (18%), bronchus intermedius (10%), left main bronchus (6%), right middle lobe (2%) and right upper lobe (2%).^[3,5]^

Symptoms of BOFs are insidious, occasionally beginning in childhood but rarely at birth. Symptoms are often not present until adulthood and even then are often intermittent.^[3]^ The duration of symptoms is variable and ranges between 6 months and 50 years before treatment is instituted.^[6]^ Symptoms include coughing, choking, haemoptysis and recurrent respiratory tract infections. The latter results in suppurative lung diseases such as lung abscesses, empyema and bronchiectasis,^[5,6]^ which can present with clubbing, basal crackles and pleural effusions. Some patients present with gastrointestinal symptoms such as dysphagia, epigastric discomfort and reflux caused by the stomach filling with air on expiration, although these are uncommon.^[3]^ Non-specific respiratory symptoms include coughing and frequent infections,^[5]^ or coughing when ingesting liquids precipitated by certain postures.^[5,6]^

Possible reasons for the delay in the onset of symptoms include the presence of an oesophageal fold that obstructs the opening of the fistula, the presence of a membrane that ruptures or a fistulous tract that is directed upwards from the oesophagus, allowing gravity to assist spasms of the smooth muscle in the fistula wall. Occasionally, patients adapt to the symptoms.^[3,6]^

Diagnosis is made by an upper gastrointestinal tract contrast study. Bronchoscopy and oesophagoscopy may demonstrate the orifice of the fistula, but it is usually small and recognisable only when the site is known. Infusing saline into the trachea with positive pressure ventilation while observing for bubbles at the site of the fistula can aid in the diagnosis. In addition, instilling methylene blue or a nontoxic dye into the oesophagus during bronchoscopy or in the trachea during oesophagoscopy may also delineate the fistula.^[2]^ A CT scan or magnetic resonance imaging can reveal the fistula.

Definitive treatment is surgical closure of the fistula, with excision of the damaged lung segments. A further approach, if thoracotomy is not feasible, is obliteration of the oesophageal end of the fistula with silver nitrate,^[3]^ biologic glue or a Celastin tube.^[2]^

This case describes an extremely rare and unusual cause of haemoptysis secondary to a type 4 congenital BOF. A congenital BOF should be suspected in patients with recurrent or persistent respiratory symptoms related to swallowing difficulties or choking in any age group or with unexplained suppurative lung diseases.
